# Does anti-malarial drug knowledge predict anti-malarial dispensing practice in drug outlets? A survey of medicine retailers in western Kenya

**DOI:** 10.1186/1475-2875-11-263

**Published:** 2012-08-06

**Authors:** Andria Rusk, Nathan Smith, Diana Menya, Andrew Obala, Chrispinus Simiyu, Barasa Khwa-Otsyula, Wendy O’Meara

**Affiliations:** 1 Duke Global Health Institute, Trent Hall, Durham, North Carolina, USA; 2 Moi University School of Medicine, Nandi Rd, Eldoret, Kenya; 3 Webuye Demographic Surveillance Site Scientific Steering Committee, Eldoret, Kenya; 4 Moi University School of Public Health, Nandi Rd, Eldoret, Kenya; 5 Duke University School of Medicine, Division of Infectious Diseases, Durham, North Carolina, USA

**Keywords:** AMFm, Antimalarial, Retail shops, Medicine outlets, Training

## Abstract

**Background:**

Malaria is a major cause of morbidity and mortality in Kenya, where it is the fifth leading cause of death in both children and adults. Effectively managing malaria is dependent upon appropriate treatment. In Kenya, between 17 to 83 percent of febrile individuals first seek treatment for febrile illness over the counter from medicine retailers. Understanding medicine retailer knowledge and behaviour in treating suspected malaria and dispensing anti-malarials is crucial.

**Methods:**

To investigate medicine retailer knowledge about anti-malarials and their dispensing practices, a survey was conducted of all retail drug outlets that sell anti-malarial medications and serve residents of the Webuye Health and Demographic Surveillance Site in the Bungoma East District of western Kenya.

**Results:**

Most of the medicine retailers surveyed (65%) were able to identify artemether-lumefantrine (AL) as the Kenyan Ministry of Health recommended first-line anti-malarial therapy for uncomplicated malaria. Retailers who correctly identified this treatment were also more likely to recommend AL to adult and paediatric customers. However, the proportion of medicine retailers who recommend the correct treatment is disappointingly low. Only 48% would recommend AL to adults, and 37% would recommend it to children. It was discovered that customer demand has an influence on retailer behaviour. Retailer training and education were found to be correlated with anti-malarial drug knowledge, which in turn is correlated with dispensing practices. Medicine retailer behaviour, including patient referral practice and dispensing practices, are also correlated with knowledge of the first-line anti-malarial medication. The Kenya Ministry of Health guidelines were found to influence retailer drug stocking and dispensing behaviours.

**Conclusion:**

Most medicine retailers could identify the recommended first-line treatment for uncomplicated malaria, but the percentage that could not is still too high. Furthermore, knowing the MOH recommended anti-malarial medication does not always ensure it is recommended or dispensed to customers. Retailer training and education are both areas that could be improved. Considering the influence that patient demand has on retailer behaviour, future interventions focusing on community education may positively influence appropriate dispensing of anti-malarials.

## Background

Malaria is a major cause of morbidity and mortality, with infection by *Plasmodium falciparum* responsible for approximately 515 million clinical infections annually [1]. An estimated 0.5 to 2.5 million deaths result from these infections, with over 90% of these borne by Africa alone [2]. In Kenya, it is the fifth leading cause of death in both children and adults [3].

In response to the high levels of parasite resistance to sulphadoxine-pyrimethamine (SP), the Kenyan Ministry of Health changed its first-line anti-malarial policy for treating uncomplicated malaria to artemether-lumefantrine, an artemisinin-based combination therapy (ACT), in 2006. With this new treatment recommendation in place, it became vital that development of drug resistance to this new therapy was delayed as long as possible, and that anti-malarial drug providers adhered to the new recommendation, discontinued the use and prescription of ineffective treatments, and that access to ACT was broadened to reach as many infected individuals as possible.

In sub-Saharan Africa, studies have shown that most families first seek treatment for mild febrile illnesses in the retail sector rather than through public health services [4,5]. Particularly in populations with inadequate access to health services, self-medicating with drugs purchased at retail locations is a common practice [6-9]. To increase access to ACT, efforts are being invested in interventions targeting retail drug locations.

In August of 2010, Kenya was one of six countries to launch Affordable Medicines Facility-malaria (AMFm), a global subsidy program aimed at reducing the price of, and improving access to, ACT. One goal of AMFm is to increase availability of the Ministry of Health recommended first-line anti-malarial treatment while decreasing the use of SP and other anti-malarials for which there is known drug resistance. Although AMFm has expanded access to affordable ACT in the private sector [10], drug retailers have been found to continue prescribing outdated anti-malarials even with knowledge of current recommended drugs. In two previous studies, one conducted in rural Tanzania and the other in Kenya, drug retailers who knew the recommended first-line treatment for malaria were found to continue dispensing and selling ineffective medications [11,12].

This study was conducted in November 2010 to survey all private medicine retailers, including shops selling primarily medicines and privately owned clinics, in the Webuye Health and Demographic Surveillance Site (WHDSS) in the Bungoma East District of western Kenya. The study focused on evaluating medicine retailer knowledge of anti-malarials, specifically the first-line recommended anti-malarial medication, as well as dispensing and treatment practices. An additional goal of the study was to discover if medicine retailers were aware of the change in Ministry of Health guidelines regarding first-line anti-malarial recommendations, and if this knowledge had an impact on dispensing practices. The location of a retail business, in an urban or rural setting, as well as the type of retail business, as a shop or a private clinic, were also examined to better understand the determinants of medicine retailer knowledge and behaviour.

## Methods

### Study area and sample

The study was conducted in the Webuye Health and Demographic Surveillance Site (WHDSS) in the Bungoma East District of Kenya’s Western Province. Kenya has a population of 39.8 million people according to the 2009 census, 78% of whom live in rural areas [13]. The Bungoma East District is located approximately 380 kilometers west of Nairobi, the capital of Kenya. The WHDSS is home to approximately 70,000 residents, and is described in detail elsewhere [14]. The primary economy is subsistence farming, with roughly 1,500 residents employed by a local sugar-processing factory, and others growing sugar cane to sell to the factory. There is a small peri-urban centre just outside the DSS boundaries where the district hospital is located. The malaria burden in the WHDSS is particularly high, despite the varied transmission rates of the Western Province [15]. A study conducted in 1998 in western Kenya found *P. falciparum* parasites in 55.4% of asymptomatic children in the wet season and 44% of children in the dry season [16].

The healthcare services within the WHDSS are provided by both the public and the private health sector. The public sector is made up of four government-owned health facilities: one district hospital, one health centre, and two medicine dispensaries. In addition to these is one faith-based hospital that offers healthcare services. The private health sector is made up of many retail businesses, including privately owned pharmacies, drug outlets, traditional healers, and herbalists.

The study population included all drug-selling locations that are privately owned, whether they self-identify as pharmacies, chemists, specialized medicine retailers, or private clinics, that are located within or are accessible to those living in the WHDSS, up to or within 5 kilometers of the WHDSS borders [10]. These locations were identified by a team of 10 fieldworkers each assigned a specific region to cover on motorbike. Fieldworkers started in the market centre and identified each outlet, taking down the name and GPS coordinates of each. At each outlet, fieldworkers asked outlet staff if there were any additional outlets in the area. Fieldworkers continued visiting outlets and inquiring after additional outlets until no new outlets were identified. Inclusion criteria comprised of locations selling anti-malarials, including privately owned clinics, medicine retail shops, chemists, privately owned pharmacies, and agrovets. Retail drug outlets were defined as shops that are privately owned, and sell medication (specifically anti-malarials), and may or may not be licensed to sell these medications. Agrovets were defined as a sub-class of those drug outlets that sell medications for human ailments, often including anti-malarials, as well as products for veterinary use. Exclusion criteria included public health facilities, general shops that sold mostly consumables and only a few medications, and refusal to participate in the survey.

### Data collection

An interviewer-administered survey tool was used to collect data on medicine retailer knowledge of the Ministry of Health recommended first-line anti-malarial drug treatment for uncomplicated malaria, self-reported actions regarding diagnosis and dispensing practices, and responses to scenarios involving customer interactions. The scenarios included mothers inquiring after medication for a sick child with specific symptoms, or requesting specific medication. Basic demographic data on medicine retailers were included. Respondents’ health qualification was categorized by type and by level. Responses of health qualification types were pharmacy-trained, including pharmacists, pharmacy technologists, and pharmacy assistants, nurse or midwifery trained, including nurses, nursing assistants, and midwives, and others, including clinical officers and laboratory technicians. Responses of health qualification level were professional-level training, including pharmacists, nurses, and clinical officers, and assistant-level training, including pharmacy assistants and nursing assistants.

Details on the outlet location were also included in the survey. Outlets located within Webuye Town were classified as urban locations, while locations outside of Webuye Town were considered rural. Outlets were classified as clinics if they self-identified as clinics by business name. All others, such as pharmacies, chemists, and *duka la dawa* (medicine shop) were classified as ‘shops’.

The survey included components from two previous questionnaires used in retail outlet studies [17,18]. Fieldworkers were trained on the survey tool, which was piloted outside the study area. The fieldworkers visited each location that had originally been identified during the canvassing activity. They interviewed all the employees in each outlet who dispensed medicines to customers. All study participants provided verbal consent before participating in the survey.

### Data entry and analysis

Fieldworkers asked questions and recorded responses on paper questionnaires. All collected data was double entered into a Microsoft Access database and confirmed, with discrepancies clarified by consulting the original survey forms. Data was exported to and analyzed using Stata v11. Unadjusted odds ratios were used to explore factors related to anti-malarial drug knowledge and practices. We considered p values of less than 0.05 to be statistically significant. Adjusted odds ratios were used when looking at the relationship between recommending appropriate treatment in children and referral behaviour, correcting for anti-malarial drug knowledge. Exact logistic regression was used for comparisons with fewer than nine observations in any category.

### Ethical considerations

Ethical approval for the research was granted from ethical review boards at Moi University Institutional Research and Ethics Committee in Eldoret, Kenya and the Duke University Institutional Review Board in Durham, North Carolina. Additional approval was also sought from the Webuye HDSS site authorities, community chiefs, assistant chiefs, and village elders from the study area.

## Results

### The study community

There were 117 individuals from 97 different outlets that participated in the survey. Of these, two individuals were excluded from analysis on the basis that they did not dispense medications and could not comment on anti-malarial dispensing practices. Nearly all the outlets included in the survey were in a rural setting (94%), and were classified as shops (87%) rather than clinics (Table 1).

**Table 1:** Characteristics of surveyed outlets

**Variables**	**N = 97**	**%**
**Type of retail outlet**		
Shop	n = 84	87%
Clinic	n = 13	13%
**Location of retail outlet**		
Urban	n = 6	6%
Rural	n = 91	94%
**Operating hours**		
< 8 hours per day	n = 3	3%
8 - 10 hours per day	n = 54	56%
> 10 hours per day	n = 39	40%
Open on Saturday	n = 82	85%
Open on Sunday	n = 41	42%
**Number of staff -- Mean (min - max)**	1.64 (1–6)	
**Number who dispense -- Mean (min - max)**	1.35 (1–3)	

### Characteristics of medicine retailers

Of the 115 respondents included in the analysis (Table 2), 71% were female, and 83% were under the age 40, with the largest proportion of respondents (n = 52) between the ages of 30 and 40. The majority of retailers surveyed had reached an education level above secondary school (67%), with fewer having just completed secondary school (30%), and the smallest proportion having finished only primary school (2%, n = 2).

**Table 2:** Background information on the surveyed population, individual level variables, Western Province, Kenya 2010

**Variables**	**N = 115**	**%**
**Age**		
Age 15-20	n = 1	1%
Age 20-30	n = 43	37%
Age 30-40	n = 52	45%
Age 40-50	n = 9	8%
Age 50-60	n = 6	5%
Age 60+	n = 2	2%
**Female Ratio**	71%	
**Health Qualification**		
Pharmacist	n = 25	22%
Pharmacy Technologist	n = 10	9%
Pharmacy Assistant	n = 10	9%
Clinical Officer	n = 1	1%
Nurse	n = 38	33%
Nurse Assistant	n = 9	8%
Laboratory Technician	n = 1	1%
Other	n = 6	5%
None	n = 15	13%
**Education**		
Completed primary	n = 2	2%
Completed Secondary	n = 35	30%
Some or completed above secondary	n = 77	67%
**Anti-malarial Training**		
Attended malaria workshop	n = 46	40%
Attended malaria workshop after 2006	n = 34	30%
Received drug information from MOH	n = 94	82%

Over 83% of respondents had received health-related training, with most of these having been trained in pharmacy (40%) or in nursing/midwifery or as a clinical officer (42%). 18% of the survey respondents had no relevant health training. These results are incongruous with the self-reported education levels, since 82% of those surveyed reported a health training in either pharmacy, nursing/midwifery, or as a clinical officer but only 67% had received an education above secondary school.

Study findings showed a gender disparity in the health training results. Male retailers were nearly three times more likely than female retailers to have no related health training (OR = 3.77, 95% CI 1.41 – 10.10; p = 0.008). Men were also less likely to be trained in nursing or midwifery than women (OR = 0.22, 95% CI 0.08 – 0.59; p = 0.003).

### Anti-malarial drug knowledge

In the survey, each respondent was asked an open-ended question to identify which anti-malarial medication is recommended by the Ministry of Health to treat uncomplicated malaria. Most of the population surveyed correctly identified the first-line treatment (65%), but the odds of knowing the recommended treatment were higher amongst participants with health training (OR = 3.26, 95% CI 1.23 – 8.63; p = 0.017). Seventy-one percent of those with health training correctly identified the recommended anti-malarial medication, but less than half the retailers without any health training identified the recommended first-line anti-malarial (Table 3). There was no difference in the percent of the nursing/midwifery trained who identified AL as the first-line therapy compared to those with pharmacy training, and both were more likely to identify AL when compared to retailers without training (OR = 3.28, 95% CI 1.12 – 9.65; p = 0.031 pharmacy trained compared to the untrained; OR = 3.24, 95% CI 1.12 – 9.39’ p = 0.031 nurse/midwifery trained compared to the untrained).

**Table 3:** Association of medicine retailer characteristics with correctly identifying the MOH recommended first-line anti-malarial therapy

	**N**	**% (n)**	**Unadjusted odds ratio and (95% confidence interval; P value)**
**Age**			
Under 30	44	77% (34)	1 (reference)
Between 30 and 40	52	52% (27)	0.32 (0.13 - 0.77; 0.012)*
Over 40	17	82% (14)	1.37 (0.33 - 5.75; 0.665)
**Gender**			
Female	82	66% (54)	1 (reference)
Male	32	66% (21)	0.99 (0.42 - 2.34; 0.982)
**Health qualification type**			
Untrained	21	43% (9)	1 (reference)
Pharmacy	45	71% (32)	3.28 (1.12 - 9.65; 0.031)*
Nurse/Midwife	48	71% (34)	3.24 (1.12 - 9.39; 0.031)*
**Health qualification level**			
Untrained	21	43% (9)	1 (reference)
Assistant level	29	76% (22)	4.14 (1.25 - 14.09; 0.021)*
Professional level	64	69% (44)	2.93 (1.07 - 8.08; 0.037)*
**Education level**			
Secondary or below	37	62% (23)	1 (reference)
Above secondary	77	68% (52)	1.27 (0.56 - 2.87; 0.572)

* denotes statistical significance.

The level of health training, whether a retailer was trained at an assistant level, such as a nursing assistant or pharmacy assistant, rather than a professional, such as a pharmacist, clinical officer, or nurse, was not a significant predictor of identifying the MOH recommended first-line anti-malarial. A slightly higher proportion of assistants correctly identified the first-line anti-malarial, at 76% (n = 22), compared to professionals, at 69% (n = 44), although this comparison did not reach statistical significance (OR = 1.43, 95% CI 0.525 – 3.89; p = 0.485).

Assistants were less likely to have attended a workshop on malaria treatment than professionals (OR = 0.27, 95% CI 0.097 – 0.751; p = 0.012). However, those who attended a workshop on malaria were not more likely to know the correct first-line anti-malarial, even if that workshop was after the change to AL as the first-line therapy in 2006 (Table 4a).

**Table 4:** a: Relationship between shop worker knowledge of Ministry of Health recommended first line anti-malarial therapy and malaria training and dispensing practices

	**n**		**% who correctly identified the firstline anti-malarial therapy**	**Unadjusted odds ratio (95% confidence interval; P value)**
**Malaria training**				
Attended malaria workshop				
	Yes	46	65%	0.94 (0.42 - 2.07; 0.873)
	No	66	67%	Ref
Attended malaria workshop after 2006				
	Yes	34	62%	0.32 (0.06 - 1.71; 0.184)
	No	12	83%	Ref
Received drug information from MOH				
	Yes	94	69%	0.96 (0.85 - 1.08; 0.483)
	No	19	53%	Ref
**Dispensing Practices**				
Recommended AL for uncomplicated malaria in children under 5				
	Yes	43	74%	2.41 (1.01 - 5.76; 0.048)*
	No	53	55%	Ref
Recommend AL for uncomplicated malaria in adults				
	Yes	55	76%	3.55 (1.49 - 8.47; 0.004)*
	No	42	48%	Ref
**Table **4**b: Relationship between shop worker recommendation of AL to children under 5 and their response to clinical scenarios**				
		**n**	**% who would recommend AL to children under 5**	**Unadjusted odds ratio and (95% confidence interval; P value)**
Paediatric Case Management**				
Refer pediatric patients rather than treat				
	Yes	43	49%	2.45 (1.04 - 5.79; 0.041)*
	No	65	31%	Ref
Refuse Fansidar to a mother requesting it				
	Yes	94	67%	3.59 (1.08 - 11.90; 0.036)*
	No	19	58%	Ref

* denotes statistical significance.

**self-reported responses to open-ended clinical scenario.

Level of education was not related to anti-malarial dug knowledge. Sixty-two percent of those with a secondary education or below identified AL as the MOH recommended first-line therapy for malaria, and 68% of those with above a secondary education identified the first-line anti-malarial medication.

### Anti-malarial dispensing practices

In the survey, each respondent was asked an open-ended question to identify which anti-malarial medication they would recommend to an adult or a pediatric client with uncomplicated malaria. Retailers were not asked to specify the dosages. In both cases, knowing the MOH recommended anti-malarial medication was found to be a strong predictor of recommending this medication (Table 4a).

Health-related qualification was also a significant predictor of recommending the proper anti-malarial treatment.

Pharmacy-trained retailers had the highest proportion of those who recommended AL as the first-line anti-malarial treatment to adults when compared to untrained retailers (OR = 4.50, 95% CI 1.263 – 16.036; 0.020). However, training in nurse-midwifery was not a statistically significant predictor of recommending AL to adults (OR = 2.30, 95% CI 0.67 – 7.86; 0.184). For paediatric clients, a higher proportion of those trained in nursing or midwifery would recommend AL (44%) compared to those trained in pharmacy (38%) and those without training (24%), although these comparisons did not reach statistical significance.

The level of health training was also associated with recommending the appropriate first-line anti-malarial drug, particularly in adults. Those with a professional level of training were more than three times more likely to recommend this treatment to adults when compared to the untrained (OR = 3.89, 95% CI 1.16 – 13.03; p = 0.027). There was a general trend of increasing proportion of respondents correctly recommending AL to both adults and children as the level of training increased, except that assistants seemed to have a slightly higher proportion recommending AL to children than professionals. However, this did not reach statistical significance.

Recommending the current first-line anti-malarial to either adult or pediatric clients was not associated with having attended a workshop on malaria or anti-malarial drugs (Table 4), regardless of whether the workshop was held before or after the change to AL in 2006. The same is true for medicine retailers having received information on anti-malarial drugs from the Ministry of Health.

### Retailer actions and behaviours

Study participants were asked to respond to a scenario in which a mother requests Fansidar tablets, an outdated anti-malarial medication containing sulphadoxine and pyrimethamine (SP), for a sick child. How a participant would react in this situation was recorded as an open-ended response. Thirty-five percent of respondents said they would give the mother different medicine, though these responses did not determine whether that medicine would be an anti-malarial or not. Twenty-five percent of respondents would refer the mother and child to a heath facility or laboratory for testing, 17% would dispense Fansidar to the mother, 14% would request more history, and 4.5% each would either give the mother a non-malaria drug or would tell her they do not have Fansidar.

The responses to the scenario formed two groups – those who would dispense SP that the mother requested, and those who would refer the child or ask for more information about the child’s condition. It was found that retailers in the latter group were 2.5 times more likely to have reported AL as the treatment they would recommend to children with uncomplicated malaria (OR = 2.45, 95% CI 1.04 – 5.80; 0.041) (Table 4). Even when controlling for whether a retailer knew or did not know the MOH-recommended first-line anti-malarial, referral practice remains correlated with recommending appropriate treatment for children (Adjusted OR = 2.50, 95% CI 1.04 – 6.02; 0.041).

Choosing to refer rather than dispense SP was associated with knowing the appropriate first-line anti-malarial medication, but there was still a high percentage of retailers who knew the MOH recommended anti-malarial and would dispense SP to a mother who requests it. Of those who would give SP in this scenario, 58% of them had previously reported AL as the MOH recommended first-line anti-malarial treatment.

Whether a retailer works in a shop or a clinic is associated with dispensing SP to mothers who request it. Sixty-three percent of those working in shops would withhold SP, while 19% would dispense it to their customer. Of those working in a clinic, 92% would withhold SP in favor of further examining the patient, or referring to a laboratory or health facility, and none would dispense SP to their customer.

Retailer motivations behind choosing which anti-malarials to stock and which to dispense were also correlated with recommending the first-line anti-malarial to children. Participants were asked an open-ended question regarding how they decide what malaria medications to carry in their outlets. Those who said they stock according to the anti-malarials recommended by the Ministry of Health (n = 24) were 6 times more likely to correctly identify the MOH-recommended anti-malarial for children under 5 than those who stock according to what their customers demand (n = 74; OR = 6.00, 95% CI 1.90 – 18.99; 0.002). However, the motivations behind drug stocking were not correlated to recommending the appropriate treatment in adults.

Participants were also asked an open-ended question regarding how they choose which anti-malarial to dispense to a customer when they have more than one anti-malarial available. Those who would dispense according to MOH guidelines (n = 11) were seven times more likely to recommend the appropriate first-line treatment to children than those who dispense according to customer demands (n = 41; OR = 7.00, 95% CI 1.24 – 39.49; 0.027). Dispensing practice was not correlated with whether they recommend correctly for adults.

To understand if customer pressure could explain this gap between anti-malarial knowledge and dispensing practices, additional analyses were conducted on the relationship between medicine retailers and their customers. However, it was found that 98% of all retailers surveyed said they provide advice outside the customers’ demands, 95% suggest specific medicines to their customers, and 89% said their customers would take this advice and purchase the medication they recommend. Very few retailers were found to act solely on customer demand. It was, therefore, difficult to isolate the effect of customer demand on dispensing practices.

## Discussion

Retail medicine outlets are an important source of treatment for febrile illness, including dispensing anti-malarials. Self-medication with drugs purchased at retail outlets is a common practice [6-9,19], and medicine retailers have an important role in the diagnosis and treatment of their customers [20]. It is important to understand the determinants of medicine retailer dispensing and treatment practices if malaria case management is to be improved.

This study explored drug retailer characteristics, level of training, level of education, anti-malarial drug knowledge, and dispensing practices in rural western Kenya. In this study population there was a high proportion of medicine retailers who reported having an education at or below secondary school, 33%. This finding is in alignment with evidence from previous studies that also found education levels to vary across retailers [21,22]. However, where these studies found the proportion of participants with health-related training to be between 20 and 26%, over 80% of the participants in this study reported health-related training.

In concurrence with previous studies on medicine retailer anti-malarial drug knowledge [5,11], this study found that most retailers were aware of the current recommended treatment for uncomplicated malaria. Sixty-five percent of participants in the study population identified the Kenya Ministry of Health recommended first-line anti-malarial. Additionally, participants who correctly identified the current treatment for malaria recommended by the MOH were found to be more likely to recommend that treatment to both adult and pediatric clients. This suggests that the change in the recommended anti-malarial treatment from SP to AL made in 2006 by the MOH has had an impact on retailer anti-malarial knowledge as well as dispensing practices.

This study also found that a medicine retailer’s training is a significant determinant of their reported anti-malarial drug knowledge. Having a health-related qualification was a significant predictor of identifying the current first-line treatment for malaria when compared to having no qualification, or qualification in a field unrelated to health, indicating that it is not professional training alone that improves anti-malarial drug knowledge, but pertinent and relevant training. The type of health training, be that in the field of pharmacy or of nursing, was not found to be a determinant of identifying the recommended first-line anti-malarial medication. However, pharmacy-trained retailers were more likely to recommend appropriate treatment for adults, and nurse or midwifery-trained retailers were more likely to recommend the appropriate treatment for children. This could be a result of the curriculum behind these types of trainings, or the previous work experience common to each field. Both pharmacy and nursing/midwifery-trained retailers were significantly more likely to have appropriate knowledge and dispensing practices than the untrained, further underscoring the need for medicine retailers to have at least a basic training in a health-related field.

Even though retailer training is an important factor in knowing the current recommended first-line anti-malarial treatment, and this knowledge increases the likelihood of a retailer dispensing medication appropriately, there remained disappointingly low proportions of staff who would recommend appropriate treatment. Of those who identified the current first-line anti-malarial, only 56% would recommend AL to adults, and only 43% would recommend it to children. This finding is in keeping with evidence reported in previous studies that found the proportion of appropriate retailer behaviour to be low considering its importance for effective malaria control [5,20,21,23] and that retailers often dispense inappropriately despite having accurate treatment knowledge. This suggests that there are determinants to retailer behaviour, as it specifically relates to dispensing practices, which are stronger than the influence of retailer knowledge.

In examining treatment practices through the use of scenarios, 17% percent of respondents would dispense an ineffective medicine, SP, to a mother who specifically requested that medicine. Of these retailers, 58% of them had identified AL as the MOH recommended first-line treatment for malaria. Participants who refer a sick child rather than dispense at the mother’s request are more likely to recommend the appropriate anti-malarial medications to children than those who would treat the child and not refer.

Customer demand is potentially a strong driving force for dispensing practices. Thirty-five percent of participants said that they would dispense malaria medicine according to what customers demanded, the most frequently cited reason for choosing which medication to dispense. This finding is consistent with that of Okeke and colleagues in their study on treatment practices of drug vendors in southeast Nigeria [8]. They found that 61.5% of those surveyed would dispense medications according to what customers demanded. Participants in their study would dispense anti-malarial drugs according to what their customer requested, or could afford, even if this action was contrary to what they knew to be appropriate treatment for malaria. Given that the majority of retailers reported providing advice outside of customer demand, but that customer demand is a significant factor in stocking and dispensing practices, future research from both the customer and retailer perspectives would be useful in understanding this dynamic.

This finding is consistent with study findings regarding stocking and dispensing practices to be related to anti-malarial knowledge. Retailers who reported stocking and dispensing drugs based on Ministry of Health guidelines are more likely to identify the recommended malaria treatment than retailers who dispense according to the demands of their customers. Retailers who reported stocking their drugs according to MOH guidelines are more likely to know the current first-line anti-malarial than retailers who stock according to customer demand. This is further evidence that Ministry of Health guidelines have an impact on medicine retailer treatment and dispensing practices, but even though knowing the MOH guidelines influences stocking and dispensing practices, still too few retailers adhere to these recommendations.

There was an inconsistency between participants’ self-reported health training and education levels, since 82% of the study participants reported having health training in pharmacy or nursing/midwifery, but only 67% of the population reported an education level above secondary school. These results could be explained by either the participants misunderstanding the health training survey question, or that they self-identify with a certain health title that comes from holding that occupation rather than from having formal training. A similar dichotomy was discovered in a recent qualitative investigation that studied a similar population in the same area [20]. It was the opinion of the local research team that medicine retailers, particularly in the rural areas, are referred to by the local community as “pharmacists” or “nurses” because these are the roles they fill in the community, even if those individuals do not hold official health qualifications in that field. It was conjectured that these retailers may self-identify in accordance with these roles. If some participants who self-identified as nurses or pharmacists actually lacked formal training, this could explain why relatively low proportions of trained personnel knew and recommended the first-line therapy. If this is the case, it would be expected that the association between training and knowledge of effective anti-malarials would be stronger than was observed.

A strong gender divide was also revealed in this study, indicating that male and female retailers are consistently dissimilar in whether they have health training and the type of health training they have, and that these factors may influence the additional differences in patient treatment practices. The men surveyed in this study were nearly four times more likely than women to be untrained and are less likely to be trained in nursing or midwifery, a health field associated with higher proportions of appropriately recommending treatment in children.

All data were self-reported actions, not directly observed behaviours, and are, therefore, subject to reporting bias. Particularly in reference to training, it is possible that many respondents over-reported their qualifications. The interviewer did not directly observe training credentials and licenses. The study was also conducted over one geographic area, which may limit generalizability of study findings.

## Conclusions and recommendations

Malaria remains a major challenge to the health and well-being of the people of Kenya, and is still the nation’s fifth leading cause of death of both children and adults [3]. The appropriate use of anti-malarials and the fast and accurate treatment of malaria cases are both critical to malaria control [24]. Retail drug outlets are often the first point of care sought by patients, particularly for children with febrile episodes [9]. Drugs purchased at retail outlets are often used to self-treat [6-9,19] and drug retailers have been found to have a role in the diagnosis of their customers as well as the selection of medications purchased [20].

The relationship retailers have with their customers positions them well to increase the rate of appropriate treatment for malaria and improve malaria case management. It is then of utmost importance to target training and education interventions in retail drug outlets and to inform such interventions with the evidence found in medicine retailer behaviour and practice studies. The findings of this study are confirmation to most previous studies. Most retailers are aware of the recommended first-line treatment for malaria, but the percentages that are not aware is far too high, and reported adherence to that knowledge is far too low. While it is encouraging to see evidence that Ministry of Health guidelines for appropriate anti-malarials are well known amongst retailers, it is important to recognize that significant improvements to retailer knowledge and behaviour need to be realized before malaria control can be improved in this area. Medicine retailer treatment and dispensing practices are a vital part of appropriate management of malaria. Knowing the right anti-malarial medication is an important first step, but adhering to that knowledge influences patient outcomes directly.

Well-designed interventions should focus on the large population of untrained retailers, as their outcomes in terms of knowledge and dispensing practices are poorest. It is important for medicine retailers to have health-related training, that their training is current, and that they be given specific information regarding the appropriate treatment of malaria in children. Interventions should also have educational components focused on patient management, including referral practice for patients under five years of age. Previous studies have documented the success of education efforts at the retail provider level. Marsh and colleagues [25] found that educating retail drug outlet workers in rural Kenya increased the frequency and accuracy of anti-malarial drug advice given to customers, had a positive effect on customer adherence to recommended dosages, and improved anti-malarial drug access to children.

There is an opportunity for future interventions to leverage the influence customer demand has on retailer behaviour by focusing education efforts on patient communities. Specifically targeting mothers may additionally improve outcomes for children, and help increase awareness of AL as the first-line treatment for malaria in both children and adults.

## Competing interests

The authors declare that they have no competing interests.

## Authors’ contributions

AR performed data analysis and drafted the first version of the manuscript. NS participated in study design, led development of the data collection tool, collected the data, and provided revisions to the manuscript. DM participated in developing the data collection tool, and provided revisions to the manuscript. AO participated in developing the data collection tool. CS participated in data collection. BO participated in developing the data collection tool. WPO conceived the study, participated in study design and developing the data collection tool, performed analysis, and provided revisions to the manuscript. All authors read and approved the final manuscript.
